# Meat intake and risk of mortality and graft failure in kidney transplant recipients

**DOI:** 10.1093/ajcn/nqab185

**Published:** 2021-06-05

**Authors:** M Yusof Said, Angelica Rodriguez-Niño, Adrian Post, Joelle C Schutten, Lyanne M Kieneker, Antonio W Gomes-Neto, Marco van Londen, Maryse Cj Osté, Karin J Borgonjen-van den Berg, Ilja M Nolte, Else van den Berg, Pim de Blaauw, Jennifer van der Krogt, M Rebecca Heiner-Fokkema, Gerjan Navis, Benito A Yard, Stephan Jl Bakker

**Affiliations:** Department of Internal Medicine, Division of Nephrology, University Medical Center Groningen, University of Groningen, Groningen, The Netherlands; Department of Internal Medicine, Division of Nephrology, University Medical Center Groningen, University of Groningen, Groningen, The Netherlands; Vth Department of Medicine (Nephrology/Endocrinology/Rheumatology), University Medical Center Mannheim, University of Heidelberg, Mannheim, Germany; Department of Internal Medicine, Division of Nephrology, University Medical Center Groningen, University of Groningen, Groningen, The Netherlands; Department of Internal Medicine, Division of Nephrology, University Medical Center Groningen, University of Groningen, Groningen, The Netherlands; Department of Internal Medicine, Division of Nephrology, University Medical Center Groningen, University of Groningen, Groningen, The Netherlands; Department of Internal Medicine, Division of Nephrology, University Medical Center Groningen, University of Groningen, Groningen, The Netherlands; Department of Internal Medicine, Division of Nephrology, University Medical Center Groningen, University of Groningen, Groningen, The Netherlands; Department of Internal Medicine, Division of Nephrology, University Medical Center Groningen, University of Groningen, Groningen, The Netherlands; Department of Human Nutrition and Health, Wageningen University, Wageningen, The Netherlands; Department of Epidemiology, University of Groningen, Groningen, The Netherlands; Department of Internal Medicine, Division of Nephrology, University Medical Center Groningen, University of Groningen, Groningen, The Netherlands; Department of Laboratory Medicine, University Medical Center Groningen, University of Groningen, Groningen, The Netherlands; Department of Laboratory Medicine, University Medical Center Groningen, University of Groningen, Groningen, The Netherlands; Department of Laboratory Medicine, University Medical Center Groningen, University of Groningen, Groningen, The Netherlands; Department of Internal Medicine, Division of Nephrology, University Medical Center Groningen, University of Groningen, Groningen, The Netherlands; Groningen Kidney Center, Groningen, The Netherlands; Vth Department of Medicine (Nephrology/Endocrinology/Rheumatology), University Medical Center Mannheim, University of Heidelberg, Mannheim, Germany; Department of Internal Medicine, Division of Nephrology, University Medical Center Groningen, University of Groningen, Groningen, The Netherlands; Groningen Kidney Center, Groningen, The Netherlands

**Keywords:** kidney transplantation, animal protein intake, red meat, white meat, mortality, graft failure, long-term survival, 1-methylhistidine, 3-methylhistidine

## Abstract

**Background:**

It is unknown whether meat intake is beneficial for long-term patient and graft survival in kidney transplant recipients (KTR).

**Objectives:**

We first investigated the association of the previously described meat intake biomarkers 1-methylhistidine and 3-methylhistidine with intake of white and red meat as estimated from a validated food frequency questionnaire (FFQ). Second, we investigated the association of the meat intake biomarkers with long-term outcomes in KTR.

**Methods:**

We measured 24-h urinary excretion of 1-methylhistidine and 3-methylhistidine by validated assays in a cohort of 678 clinically stable KTR. Cross-sectional associations were assessed by linear regression. We used Cox regression analyses to prospectively study associations of log_2_-transformed biomarkers with mortality and graft failure.

**Results:**

Urinary 1-methylhistidine and 3-methylhistidine excretion values were median: 282; interquartile range (IQR): 132–598 µmol/24 h and median: 231; IQR: 175–306 µmol/24 h, respectively. Urinary 1-methylhistidine was associated with white meat intake [standardized *β* (st *β*): 0.20; 95% CI: 0.12, 0.28; *P <* 0.001], whereas urinary 3-methylhistidine was associated with red meat intake (st *β*: 0.30; 95% CI: 0.23, 0.38; *P <* 0.001). During median follow-up for 5.4 (IQR: 4.9–6.1) y, 145 (21%) died and 83 (12%) developed graft failure. Urinary 3-methylhistidine was inversely associated with mortality independently of potential confounders (HR per doubling: 0.55; 95% CI: 0.42, 0.72; *P* < 0.001). Both urinary 1-methylhistidine and urinary 3-methylhistidine were inversely associated with graft failure independent of potential confounders (HR per doubling: 0.84; 95% CI: 0.73, 0.96; *P* = 0.01; and 0.59; 95% CI: 0.41, 0.85; *P =* 0.004, respectively).

**Conclusions:**

High urinary 3-methylhistidine, reflecting higher red meat intake, is independently associated with lower risk of mortality. High urinary concentrations of both 1- and 3-methylhistidine, of which the former reflects higher white meat intake, are independently associated with lower risk of graft failure in KTR. Future intervention studies are warranted to study the effect of high meat intake on mortality and graft failure in KTR, using these biomarkers.

## Introduction

Kidney transplant recipients (KTR) are at high risk of premature mortality and decline of renal function (1, 2). In KTR, high dietary protein intake has been associated with lower risk of premature mortality and graft failure through an as yet unknown mechanism (3, 4). Whether the source of dietary protein is relevant to outcome in KTR is unknown. Meat is an important source of dietary protein. Studies of 2 types of meat have been reported extensively in the literature: white and red meat. Several large cohort studies in the general population have found that high red meat intake is associated with increased risk of chronic kidney disease, kidney failure, and death (5–7). Conversely, white meat intake has been associated with lower risk of mortality in the general population (5). Currently, it is unknown whether white meat, red meat, or both are associated with long-term outcomes in KTR.

One of the challenges of estimating meat intake through a food frequency questionnaire (FFQ) is that the estimations are prone to limitations, including under- and overreporting, illiteracy, motivation requirements, recall bias, errors in portion size estimation, and socially desirable answers (8, 9). The use of meat-specific biomarkers might be a more accurate approach in estimating true meat intake. A proposed marker for white meat intake is 1-methylhistidine, which results from the metabolism of the dipeptide anserine (10, 11). Up to 90% of dietary anserine is hydrolyzed to 1-methylhistidine and excreted via urine (12). Previous studies found that both plasma and urinary 1-methylhistidine are associated with white meat intake, which comprises predominantly poultry intake (13, 14). A proposed biomarker for red meat intake is 3-methylhistidine, which is found in myosin and actin (15) and is formed after methylation of histidine moieties and released after catabolism of proteins (11, 15, 16). Thereafter, 3-methylhistidine is neither further reutilized nor metabolized but instead is excreted as 3-methylhistidine via urine (17). Skeletal muscle, being the main source of actin and myosin, is regarded as the predominant source of urinary 3-methylhistidine.

In a controlled dietary intervention study of 33 adult men and 17 adult women, Altorf-van der Kuil et al. found that urinary excretion of 1-methylhistidine (uex1MH) and 3-methylhistidine (uex3MH), respectively explained 69% and 72% of variation in total meat intake (18), making these urinary metabolites putative biomarkers of meat intake.

In the present study, we aimed to investigate the potential association of uex1MH and uex3MH with FFQ-derived estimates of meat intake in a large cohort of clinically stable KTR who were not subjected to dietary protein intake restrictions. Secondly, we aimed to prospectively study the association of uex1MH and uex3MH with long-term outcomes, i.e., mortality and graft failure, in KTR.

## Methods and Materials

### Study population

From November 2008 to March 2011, adult KTR who received a transplant ≥1 y before and had a functioning graft (i.e., not on renal replacement therapy) were invited to participate to this study, as a part of a larger prospective cohort study of KTR (TransplantLines Food and Nutrition cohort, registered at clinicaltrials.gov as NCT02811835). At the time of inclusion, all KTR were undergoing clinical follow-up at the University Medical Center of Groningen, the Netherlands. Subjects with overt congestive heart failure (New York Heart Association class 3–4), medical history of cancer other than cured skin cancer, alcohol or drug abuse, or insufficient understanding of Dutch language were excluded. KTR who signed written informed consent and had frozen urine samples available for analysis were consecutively included in the study (see **Supplemental Figure 1** for a flow diagram of participant inclusion). At measurement times, subjects were at steady state, i.e., biochemically stable and without an acute illness (e.g., infection). The study protocol was approved by the institutional ethical review board (METc 2008/186) and has been conducted in accordance with the declarations of Helsinki and Istanbul.

### Data collection

Subjects were invited to the outpatient clinic for baseline measurements and collection of blood and urine samples. Blood samples were drawn after a minimal 8-h fasting period. On the same day, 24-h urine was collected by each participant, according to a well-explained protocol. Urine collection was under oil and the antiseptic agent chlorhexidine was added to the urine. Physical measurements have been described in detail previously (19–21) and were done on the same day as blood and urine collection. Questionnaires were used to obtain information on smoking and alcohol intake. We categorized smoking as never, former, or current, and alcohol intake as 0–10, 10–30, or >30 g/24 h. Diabetes mellitus was characterized by the usage of antidiabetic medication or fulfillment of the American Diabetes Association criteria of 2017: a fasting plasma glucose concentration ≥7.0 mmol/L and/or HbA1c ≥6.5%. Physical activity was measured with the Short Questionnaire to Assess Health-enhancing physical activity (SQUASH) (22). Delayed graft function was defined as need for dialysis in the first week following transplantation (23). In KTR with proteinuria at the time of baseline of the biobank and cohort study, we checked whether kidney biopsies had been performed between 2 y before and 2 y after baseline measurement. If the time between 1 y after transplantation and baseline was <2 y, we included kidney biopsies if they had been performed between 1 y after transplantation and 2 y after baseline measurement. Biopsies were performed by a trained nephrologist, prepared according to local protocol, and examined by a trained kidney pathologist.

### Dietary assessment

We used validated semiquantitative FFQs that were developed at Wageningen University and have been described in detail before (24, 25). The FFQs were distributed to the KTR to fill out at home before visit to the outpatient clinic for baseline measurements. Household units were used to express the number of serving sizes consumed (e.g., bowls or pieces) or weights. Frequency was expressed per day, week, or month. The FFQs were afterward checked by trained researchers, and patients were consulted if needed to verify answers that seemed inconsistent or if FFQs were incomplete. The questionnaire data were analyzed using the 2006 Dutch Food Composition Table (NEVO), as distributed by the Dutch Ministry of Health, Welfare, and Sport (26), to calculate intakes of energy and macro- and micronutrients. FFQs reporting energy intakes of <500 or >5000 kcal per d were regarded as unreliable and therefore excluded. Certain food items were combined to produce a composite measurement of specific meat intake, such as red meat or white meat. Red meat intake was calculated by combining the daily intakes of beef, pork, lamb, liver/kidney, and processed meat products [sausages, blind finch (a type of Dutch roulade), minced meat, bacon, and luncheon meat]. White meat intake was calculated by combining the daily intakes of chicken and turkey meats. In **Supplemental Table 1**, an overview of the specific meat intakes derived from the FFQ can be found.

In addition to the FFQ measurement of total protein intake, we also calculated total protein intake with 24-h urea excretion and protein excretion using the Maroni equation (27):

Protein intake (g/day) based on the Maroni equation

 = 0.18 × urinary urea excretion (mmol/day) + 0.19

  × body weight (kg) + urinary protein excretion (g/day) *(1)*

### Laboratory measurements

We measured concentrations of 1-methylhistidine and 3-methylhistidine from thawed 24-h urine samples using a validated UHPLC-MS/MS. The urine samples were derivatized with AccQ-Tag derivatization reagent according to the manufacturer's protocol (Waters Corporation). The derivates of 1-methylhistidine and 3-methylhistidine were separated using a Phenomenex Synergi™ column (4 µm Polar-RP 80 Å, 150 × 3 mm) and were detected using positive-ion electrospray ionization in multiple reaction monitoring mode using the following transitions: *m/z* 340.0→171.0 for 1-methylhistidine and 3-methylhistidine and 335.0→171.0 for the internal standard (13C6-, 15N3-histidine). Data were analyzed using MultiQuant MD 3.0.2 (Sciex). Two urine samples were used for assessment of intra-assay precision, and 2 others for assessment of interassay precision. The intra-assay CVs for 1-methylhistidine were 3.1% at 155 µmol/L and 4.4% at 1450 µmol/L, with interassay CVs of 12.1% at 53 µmol/L and 8.6% at 118 µmol/L. For 3-methylhistidine, the intra-assay CVs were 4.3% at 402 µmol/L and 5.4% at 604 µmol/L, and the interassay CVs were 8.4% at 99 µmol/L and 8.7% at 141 µmol/L. The accuracy was 112% for 1-methylhistidine and 109% for 3-methylhistidine compared to our reference method for amino acids on a Biochrom 30 analyzer (Pharmacia Biotech). The detection and quantification limits for 1-methylhistidine were 4.3 and 18.6 µmol/L, respectively, and for 3-methylhistidine 4.5 and 6.5 µmol/L, respectively, with a linear range up to 1000 µmol/L. Samples above this range were reported as >1000 µmol//L. Urine sample concentrations below or above the detection threshold of a specific compound were registered as at the lower or upper detection threshold, respectively. The 1-methylhistidine concentrations were below the lower detection threshold in 2 KTR and above the upper detection threshold in 8 KTR. All KTR had 3-methylhistidine concentrations within the limits of detection. Routine laboratory methods were used for other blood and urine analyses, as described earlier (19–21). Venous pH and HCO_3_^−^ were measured as described earlier (24). Urinary taurine was measured by UHPLC-MS as previously described (28). Serum iron was measured using photometry (Modular P800, Roche Diagnostics).

We calculated the estimated glomerular filtration rate (eGFR) using the Chronic Kidney Disease Epidemiology Collaboration formula with serum creatinine and cystatin C (29). Proteinuria was defined as urinary protein excretion ≥0.5 g/24 h.

### Study outcomes

Outcomes were all-cause mortality and death-censored graft failure. Graft failure was defined as return to dialysis or retransplantation. Follow-up was up to October 2015. No patients were lost to follow-up.

### Statistical analysis

Baseline data are presented as mean ± SD for normally distributed data, as median [IQR] for nonnormally distributed data, and as number (percentage) for nominal data. Since uex1MH and uex3MH had a skewed distribution, these variables were log_2_ transformed for all analyses.

We first cross-sectionally studied the separate associations of uex1MH and uex3MH (dependent variables) with basic characteristics and transplantation-related characteristics (independent variables) by performing univariable linear regression. Categorical variables were recoded into dummy dichotomous variables and analyzed together by means of multivariable linear regression.

We also cross-sectionally analyzed the associations of uex1MH and uex3MH with dietary intake estimates by first performing univariable linear regression and consecutively multivariable linear regression. In the multivariable analyses, we adjusted the associations of uex1MH and uex3MH with food intake estimates for age, sex, total caloric intake, body mass index [BMI (kg/m^2^)], and eGFR. Regression coefficients values are presented as standardized β (st β), referring to the number of SDs the dependent variable changes per SD increase of the independent variable, allowing the comparison of association strengths among different variables. As measure of variability, 95% CIs are shown in **Tables 1–3**. A paired *t*-test was employed to assess differences between FFQ-derived protein intake and Maroni-calculated protein intake.

**TABLE 1:** Associations of meat intake biomarkers with basic general characteristics^1^

			Association with log_2_-transformed biomarkers					
			uex1MH			uex3MH		
	*n*	RTR (*n* = 678)	St *β*	95% CI	*P* value	St *β*	95% CI	*P* value
General characteristics								
Age of patient, y	678	54.5 [44.8–62.9]	−0.18	−0.26, −0.10	<0.001	−0.18	−0.26, −0.11	<0.001
Male sex, *n* (%)	678	390 (57.5)	0.09	0.02, 0.17	0.01	0.47	0.41, 0.54	<0.001
Weight, kg	678	80.4 ± 16.6	0.17	0.10, 0.27	<0.001	0.43	0.36, 0.50	<0.001
BMI, kg/m^2^	678	26.6 ± 4.8	0.12	0.04, 0.19	0.002	0.22	0.15, 0.30	<0.001
Time since transplantation, y	678	5.3 [1.8–11.5]	−0.06	−0.14, 0.02	0.12	−0.12	−0.20, −0.05	0.001
Urinary protein intake biomarkers								
uex1MH, µmol/24 h	678	281.7 [132.0–597.7]	NA	NA		0.36	0.29, 0.43	<0.001
uex3MH, µmol/24 h	678	231.0 [175.4–306.3]	0.42	0.35, 0.49	<0.001	NA	NA	
Smoking behavior, *n* (%)^2^	639							
Never		267 (39.4)	Ref.			Ref.		
Ex		290 (42.8)	−0.11	−0.19, −0.02	0.01	−0.05	−0.13, 0.04	0.26
Current		82 (12.1)	−0.02	−0.10, 0.06	0.65	0.04	−0.04, 0.13	0.31
Cardiovascular parameters								
Systolic pressure, mmHg	676	136 ± 17	−0.07	−0.14, 0.01	0.08	0.04	−0.04, 0.12	0.30
Diastolic pressure, mmHg	676	83 ± 11	0.04	−0.04, 0.11	0.33	0.14	0.06, 0.21	<0.001
Total cholesterol, mmol/L	678	5.11 ± 1.12	−0.002	−0.08, 0.07	0.96	−0.10	−0.17, −0.02	0.01
HDL cholesterol, mmol/L	669	1.30 [1.10–1.60]	−0.05	−0.13, 0.02	0.18	−0.17	−0.25, −0.10	<0.001
LDL cholesterol, mmol/L	669	2.90 [2.30–3.50]	0.02	−0.05, 0.10	0.57	−0.03	−0.11, 0.05	0.44
Triglycerides, mmol/L	670	1.68 [1.25–2.29]	−0.04	−0.11, 0.04	0.35	−0.01	−0.09, 0.07	0.82
History of cardiovascular event, *n* (%)^3^	678	101 (14.9)	−0.04	−0.12, 0.03	0.27	−0.02	−0.10, 0.06	0.59
Diabetes								
Diabetes, *n* (%)^4^	678	162 (23.9)	−0.09	−0.16, −0.01	0.02	−0.04	−0.12, 0.03	0.28
Antidiabetics usage, *n* (%)	678	107 (15.8)	−0.09	−0.17, −0.02	0.02	−0.07	−0.15, 0.004	0.06
Acidosis								
Venous pH	626	7.37 ± 0.04	0.06	−0.02, 0.14	0.13	0.04	−0.04, 0.12	0.32
Venous HCO_3_^−^	626	24.6 ± 3.1	−0.04	−0.12, 0.04	0.27	−0.01	−0.09, 0.07	0.83
Inflammation								
CRP, mg/L	638	1.6 [0.7–4.5]	−0.08	−0.16, 0.002	0.04	0.02	−0.06, 0.09	0.71
Blood leucocyte, × 10^9^/L	677	8.1 ± 2.6	0.01	−0.07, 0.08	0.89	0.06	−0.01, 0.14	0.11
Urine taurine excretion, µmol/24/h	678	533 [210–946]	0.15	0.08, 0.23	<0.001	0.48	0.41, 0.54	<0.001
Serum iron, µmol/L	673	15.3 ± 6.1	0.06	−0.02, 0.14	0.12	0.06	−0.02, 0.14	0.12
SQUASH physical activity score	678	5160 [2040–8073]	0.12	0.04, 0.19	0.003	0.18	0.11, 0.26	<0.001

1 Data are presented as mean ± SD, median [IQR], or absolute number (%). Associations of biomarkers with variables were tested via univariable regression analyses of which St *β* are given, referring to the number of SD changes in the dependent variable (biomarker) per SD increment in the independent variable. CRP, C-reactive protein; HbA1c, glycated hemoglobin; KTR, kidney transplant recipient; NA, not applicable; SQUASH, Short QUestionnaire to ASess Health-enhancing physical activity; st β, standardized β coefficient; Ref, reference; uex1MH: urinary 1-methylhistidine excretion; uex3MH: urinary 3-methylhistidine excretion.

2 Categories do not sum up to 100% because of missing data [*n* = 44 (6.5%)].

3 Defined as myocardial infarction, coronary intervention (including percutaneous coronary intervention and coronary artery bypass grafting), and cerebral ischemic event (including cerebrovascular accident and transient ischemic attack).

4 Defined as blood glucose ≥7 mmol/L, HbA1c ≥6.5%, and/or use of antidiabetics.

Second, we studied prospective associations of uex1MH and uex3MH with mortality and death-censored graft failure during follow-up by performing Cox proportional hazard analyses. We used log_2_-transformed uex1MH and uex3MH to allow for interpretation of HR values per doubling of uex1MH and per doubling of uex3MH, respectively. We adjusted the associations of uex1MH and uex3MH with outcomes for potential confounders. Baseline characteristics that were significantly associated with uex1Mh and uex3MH were considered potential confounders. Model 1 included adjustments for several potential confounders, including age, sex, BMI, eGFR, proteinuria, time from transplantation to baseline visit, and FFQ-estimated energy intake. Adjustments of all subsequent models were additions to model 1 in order to prevent inclusion of too many variables per number of events. In model 2 we additionally adjusted for transplantation-related factors (postmortem donation, cold ischemia time, total dialysis time, number of previous transplantations, and primary renal disease), in model 3 for posttransplantation complications [delayed graft function, rejection after transplantation (up to baseline), CMV infection (primary or secondary)], in model 4 for immunosuppressive medication (prednisolone dosage, usage of calcineurin inhibitors, and/or proliferation inhibitors), in model 5 for alcohol intake, in model 6 for potential cardiovascular risk factors and parameters [C-reactive protein (CRP), HDL cholesterol, diastolic blood pressure, smoking behavior, diabetes mellitus, posttransplantation diabetes mellitus (PTDM, i.e., new-onset diabetes mellitus after transplantation), and SQUASH score], in model 7 for metabolic acidosis (venous pH and HCO_3_^−^), in model 8 for serum iron, and finally, in model 9 for 24-h urinary taurine excretion. Potential interactions for age, sex, BMI, eGFR, and alcohol intake, were investigated by assessing interaction terms. We performed linear spline analyses to demonstrate linearity of the prospective associations of uex1MH and uex3MH with mortality and graft failure. All data for the spline analyses were fit by a Cox proportional hazard model adjusted for age, sex, BMI, eGFR, proteinuria, time from transplantation to baseline visit to the outpatient clinic, and FFQ-estimated energy intake.

Analyses were performed with IBM SPSS statistics version 23 (2015, IBM Corp.) and the statistical software R version 3.5.1 (2018, R Foundation for Statistical Computing). *P* values ˂ 0.05 were considered statistically significant.

## Results

### General baseline characteristics, transplantation-related baseline characteristics, and urinary excretion of biomarkers

Out of 817 adult KTR, 706 signed written informed consent and 678 of these had frozen urine samples available for analyses. These 678 KTRs were included in this study. Assessments for establishing the baseline of the prospective cohort study were performed at a median time of 5.3 (IQR: 1.8–11.5) y after transplantation. Median age was 55 (IQR: 45–63) y and 58% were male. The associations of urinary excretion biomarkers with general baseline characteristics are depicted in Table 1. Median urinary excretion of 1-methylhistidine was 282 (IQR: 132–598) µmol/24 h and of 3-methylhistidine was 231 (IQR: 175–306) µmol/24 h. Uex1MH and uex3MH shared positive associations with male sex, BMI, body weight, SQUASH score, and urinary taurine excretion, and they shared inverse associations with age. Uex1MH was inversely associated with past smoking behavior, medical history of diabetes mellitus, antidiabetic medication use, and CRP concentrations. Uex3MH was positively associated with diastolic blood pressure. Furthermore, uex3MH was inversely associated with time since transplantation, total cholesterol, and HDL cholesterol (Table 1).

From the transplantation-related characteristics described in Table 2, uex1MH and uex3MH shared positive associations with living donor transplantation and high prednisolone dosage. Uex1MH was positively associated with proliferator inhibitor usage. Uex3MH was positively associated with calcineurin inhibitor usage, eGFR, and delayed graft function and was inversely associated with cold ischemia time.

**TABLE 2:** Associations of meat intake biomarkers with transplantation-related characteristics^1^

			Association with log_2_-transformed biomarkers					
			uex1MH			uex3MH		
	*n*	KTR (*n* = 678)	St *β*	95% CI	*P* value	St *β*	95% CI	*P* value
Primary renal disease, *n* (%)	678							
Primary glomerular disease		194 (28.6)	0.04	−0.07, 0.15	0.46	0.15	0.05, 0.26	0.01
Glomerulonephritis		49 (7.2)	0.03	−0.06, 0.12	0.51	0.08	−0.01, 0.17	0.08
Tubular interstitial disease		83 (12.2)	−0.01	−0.09, 0.10	0.92	0.07	−0.03, 0.16	0.16
Polycystic renal disease		139 (20.5)	0.01	−0.09, 0.11	0.82	0.02	−0.08, 0.12	0.70
Dysplasia and hypoplasia		28 (4.1)	−0.004	−0.09, 0.08	0.93	0.04	−0.05, 0.12	0.41
Renovascular disease		38 (5.6)	−0.01	−010, 0.07	0.79	0.03	−0.05, 0.12	0.44
Diabetes mellitus		34 (5.0)	−0.05	−0.13, 0.04	0.29	0.06	−0.02, 0.14	0.17
Other/unknown cause		113 (16.7)	Ref.			Ref.		
Transplantation-related characteristics								
Total dialysis time, months	669	27 [10–52]	−0.07	−0.15, 0.01	0.07	−0.02	−0.10, 0.06	0.61
HLA mismatch, *n* (%)^2^	634							
0		122 (18)	Ref.			Ref.		
1		85 (12.5)	−0.06	−0.16, 0.03	0.18	−0.03	−0.12, 0.07	0.55
2		165 (24.3)	−0.02	−0.12, 0.09	0.77	0.03	−0.07, 0.13	0.55
≥3		262 (38.6)	−0.07	−0.18, 0.03	0.18	0.10	−0.01, 0.19	0.08
Living donor transplantation, *n* (%)	678	232 (34.2)	0.08	0.01, 0.16	0.04	0.08	0.01, 0.16	0.03
Cold ischemia time, h	670	15.3 [2.8–21.0]	−0.07	−0.14, 0.01	0.09	−0.10	−0.18, −0.03	0.01
≥2 transplantations, *n* (%)	678	66 (9.7)	−0.07	−0.15, 0.01	0.05	−0.08	−0.15, −0.001	0.05
Induction immunosuppression at transplantation, *n* (%)^3^	672							
Azathioprine		26 (3.8)	0.08	−0.02, 0.19	0.13	−0.04	−0.14, 0.06	0.44
Ciclosporin A		189 (27.9)	0.03	−0.16, 0.21	0.77	−0.11	−0.29, 0.07	0.25
Tacrolimus		14 (2.1)	0.06	−0.03, 0.15	0.21	0.05	−0.04, 0.15	0.25
ATG		60 (8.8)	0.02	−0.11, 0.15	0.76	−0.01	−0.14, 0.12	0.83
OKT3 monoclonal AB^4^		16 (2.4)	−0.01	−0.11, 0.08	0.81	−0.03	−0.13, 0.06	0.51
Anti-IL2R monoclonal AB		338 (49.9)	0.12	−0.07, 0.32	0.22	0.01	−0.18, 0.21	0.90
Rituximab		2 (0.3)	0.003	−0.08, 0.08	0.94	−0.03	−0.11, 0.05	0.40
Other		27 (4.0)	Ref.			Ref.		
Immunosuppressive medication at baseline								
Prednisolone dosage, mg/24nh	678	10.0 [7.5–10.0]	0.09	0.02, 0.17	0.02	0.12	0.04, 0.19	0.002
CNI usage,^5^*n* (%)	678	381 (56.2)	0.07	−0.01, 0.14	0.09	0.09	0.02, 0.17	0.02
Proliferation inhibitor usage,^6^*n* (%)	678	567 (83.6)	0.08	0.01, 0.16	0.03	0.07	−0.01, 0.14	0.08
Rejection after transplantation (up to baseline), *n* (%)	678	177 (26.1)	0.03	−0.18, 0.35	0.52	0.04	−0.04, 0.12	0.31
PTDM, *n* (%)	678	128 (18.9)	−0.08	−0.15, 0.001	0.05	−0.02	−0.01, 0.05	0.57
Delayed graft function, *n* (%)	678	49 (7.2)	0.02	−0.05, 0.10	0.56	0.11	0.03, 0.18	0.01
Cytomegalovirus infection,^7^*n* (%)	622	173 (25.5)	−0.03	−0.11, 0.05	0.47	0.01	−0.07, 0.09	0.82
BK viral load,^8^ copies/mL	641							
Undetectable		611 (90.1)	Ref.				Ref.	
<5000		27 (4.0)	0.08	−0.003, 0.15	0.06	0.02	−0.06, 0.10	0.59
5000–10,000		1 (0.1)	−0.003	−0.08, 0.07	0.94	0.04	−0.04, 0.12	0.30
>10,000		2 (0.3)	−0.05	−0.12, 0.03	0.24	−0.02	−0.10, 0.06	0.62
Renal allograft function								
Serum urea, mmol/L	676	9.4 [7.2–13.3]	−0.05	−0.12, 0.03	0.22	−0.06	−0.13, 0.02	0.15
Serum creatinine, µmol/L	676	124 [99–160]	0.01	−0.07, 0.08	0.85	0.04	−0.03, 0.12	0.28
eGFR, mL/min/1.73m^2^^9^	663	45.4 ± 18.8	0.05	−0.02, 0.13	0.17	0.10	0.03, 0.18	0.01
Protein excretion, g/24 h	678	0.20 [0.02–0.37]	−0.03	−0.11, 0.05	0.44	0.01	−0.07, 0.09	0.87
Proteinuria (>0.5 g/24 h), *n* (%)	678	152 (22.4)	−0.02	−0.09, 0.06	0.66	−0.03	−0.11, 0.05	0.44

1 Data are presented as mean ± SD, median [IQR] or absolute number (%). Associations of biomarkers with variables were tested via univariable regression analyses of which St β are given, referring to the number of SD changes in the dependent variable (biomarker) per SD increment in the independent variable. CKD-EPI, Chronic Kidney Disease Epidemiology Collaboration; CNI, calcineurin inhibitor; eGFR, estimated glomerular filtration rate; HLA, human leukocyte antigen; KTR, kidney transplant recipient; PTDM, posttransplant diabetes mellitus; st β, standardized β coefficient; uex1MH, urinary 1-methylhistidine excretion; uex3MH, urinary 3-methylhistidine excretion.

2 Categories do not sum up to 100% because of missing data [*n* = 44 (6.5%)].

3 Categories do not sum up to 100% because of missing data [*n* = 6 (0.9%)]. All induction immunosuppression protocols included corticosteroids.

4 Muromonab-CD3.

5 For example, tacrolimus.

6 For example, mycophenolate mofetil.

7 Primary or secondary cytomegalovirus infection.

8 Categories do not sum up to 100% because of missing data [*n* = 37 (5.5%)].

9 Calculated by the CKD-EPI creatinine-cystatin C formula.

Of note, 17 KTR (2.6%) had a baseline eGFR <15 mL/min/1.73 m^2^. Kidney biopsies were performed in 19 (2.8%) subjects and were mainly performed because of unexpected renal function decline. From these biopsies, 4 (21%) showed signs of cellular rejection, 2 (11%) showed signs of humoral rejection, 2 (11%) had extensive arteriolar hyalinosis suggestive of calcineurin inhibitor toxicity, 1 (5%) had signs of BK virus infection, and 1 (5%) showed signs of focal segmental sclerosis. Some biopsies showed ≥2 of these abnormalities at the same time, while there were 9 (47%) biopsies in which no abnormalities were found.

### Dietary intakes

Information on the association of protein intake biomarkers with dietary intake patterns is shown in Table 3. Of 678 KTR, 58 (8.6%) had missing FFQ data. Maroni-calculated protein intake was 86 ± 22 g/24 h, which was close to the FFQ-derived total protein intake, 82 ± 20 g/24 h, yet significantly different (*P* < 0.001). Maroni-calculated protein intake and FFQ-derived total protein intake were significantly associated (st *β*: 0.35; 95% CI: 0.28, 0.43; *P* < 0.001).

**TABLE 3:** Meat intake biomarkers and their associations with nutritional and lifestyle variables^1^

			Association with log_2_-transformed biomarkers											
			uex1MH						uex3MH					
			Model 1			Model 2			Model 1			Model 2		
	*n*	RTR (*n* = 678)	St *β*	95% CI	*P*	St *β*	95% CI	*P* value	St *β*	95% CI	*P*	St *β*	95% CI	*P* value
Urea excretion, mmol/24 h	678	388 [309–458]	0.31	[0.24, 0.39]	<0.001	0.31	[0.23, 0.40]	<0.001	0.65	[0.60, 0.71]	<0.001	0.51	[0.45, 0.56]	<0.001
Maroni-formula protein intake, g/24 h^2^	678	86 ± 22	0.32	[0.25, 0.39]	<0.001	0.33	[0.24, 0.41]	<0.001	0.67	[0.62, 0.73]	<0.001	0.53	[0.47, 0.59]	<0.001
Alcohol intake, *n* (%)^3^	620													
0–10 g/24 h		454 (67.0)	Ref.			Ref.			Ref.			Ref.		
10–30 g/24 h		138 (20.4)	0.05	[−0.02, 0.13]	0.19	0.04	[−0.04, 0.11]	0.38	0.17	[0.08, 0.23]	<0.001	0.06	[−0.01, 0.12]	0.08
>30 g/24 h		28 (4.1)	0.05	[−0.03, 0.13]	0.21	0.04	[−0.03, 0.12]	0.30	0.10	[0.02, 0.17]	0.01	0.05	[−0.02, 0.11]	0.18
Dietary intake estimates														
Energy, kcal/d	620	2172 ± 619	0.06	[−0.02, 0.14]	0.14	NA	NA		0.24	[0.16, 0.31]	<0.001	NA	NA	
Women	270	1917 ± 475	−0.04	[−0.23, 0.10]	0.47	NA	NA		0.02	[−0.12,0.17]	0.74	NA	NA	
Men	350	2368 ± 646	0.07	[−0.03, 0.16]	0.21	NA	NA		0.12	[0.01, 0.18]	0.03	NA	NA	
Fat, g/d	620	84 [64–105]	0.04	[−0.04, 0.12]	0.27	−0.08	[−0.27, 0.10]	0.39	0.21	[0.13, 0.29]	<0.001	0.01	[−0.15, 0.16]	0.91
Saturated fat	620	30 [23–38]	0.02	[−0.06, 0.10]	0.59	−0.11	[−0.07, 0.09]	0.16	0.20	[0.12, 0.27]	<0.001	0.07	[−0.05, 0.20]	0.25
Monounsaturated fat	620	28 [21–35]	0.07	[−0.01, 0.14]	0.10	0.02	[−0.14, 0.18]	0.78	0.21	[0.13, 0.28]	<0.001	<0.001	[−0.14, 0.14]	1.00
Polyunsaturated fat	620	17 [13–23]	0.03	[−0.05, 0.10]	0.53	−0.05	[−0.17, 0.07]	0.41	0.16	[0.08, 0.23]	<0.001	−0.07	[−0.17, 0.03]	0.18
Total carbohydrate intake, g/d	620	243 [194–290]	0.04	[−0.04, 0.12]	0.35	−0.07	[−0.24, 0.10]	0.43	0.17	[0.10, 0.25]	<0.001	−0.19	[−0.33, −0.05]	0.01
Protein intake, g/d	620													
Total protein	620	82 ± 20	0.08	[0.001, 0.16]	0.05	0.17	[0.03, 0.31]	0.02	0.21	[0.13, 0.29]	<0.001	0.16	[−0.33, −0.45]	0.01
Plant protein	620	31 ± 10	0.03	[−0.05, 0.11]	0.45	−0.05	[−0.19, 0.08]	0.44	0.13	[0.05, 0.21]	0.001	−0.17	[−0.28, −0.05]	0.004
Animal protein	620	51 ± 15	0.09	[0.01, 0.16]	0.03	0.13	[0.03, 0.23]	0.01	0.19	[0.11, 0.27]	<0.001	0.16	[0.08, 0.24]	<0.001
Meat products, g/d	620													
Total meat and meat products	612	94 [72–117]	0.13	[0.05, 0.21]	0.002	0.11	[0.02, 0.19]	0.01	0.29	[0.21, 0.36]	<0.001	0.18	[0.11, 0.25]	<0.001
Red meat	612	82 [59–106]	0.06	[−0.02, 0.14]	0.13	0.03	[−0.06, 0.11]	0.52	0.30	[0.23, 0.38]	<0.001	0.19	[0.12, 0.26]	<0.001
White meat	613	11 [0–16]	0.20	[0.12, 0.28]	<0.001	0.20	[0.13, 0.28]	<0.001	−0.02	[−0.10, 0.10]	0.57	−0.01	[−0.08, 0.05]	0.73
Fish intake	612	11 [4–18]	0.13	[0.10, 0.21]	0.001	0.16	[0.08, 0.23]	<0.001	0.05	[−0.03, 0.13]	0.20	0.07	[0.01, 0.14]	0.03
Dairy, g/d	613	333 [205–480]	−0.06	[−0.14, 0.02]	0.14	−0.03	[−0.12, 0.05]	0.45	−0.06	[−0.14, 0.02]	0.16	−0.06	[−0.14, 0.01]	0.09
Of which cheese	621	30 [15–46]	−0.02	[−0.10, 0.06]	0.66	−0.02	[−0.07, 0.09]	0.71	0.05	[−0.03, 0.13]	0.24	0.03	[−0.05. 0.08]	0.35
Legumes and nuts, g/d	612	11 [4–23]	−0.04	[−0.12, 0.04]	0.32	−0.05	[−0.08, 0.08]	0.26	0.02	[−0.06, 0.10]	0.69	−0.02	[−0.09, 0.05]	0.55
Vegetables, g/d	612	106 [69–149]	−0.02	[−0.10, 0.06]	0.64	0.03	[−0.08, 0.08]	0.48	−0.06	[−0.14, 0.02]	0.14	0.004	[−0.06, 0.07]	0.91
Fruit, g/d	611	123 [66–232]	−0.04	[−0.12, 0.04]	0.30	0.01	[−0.07, 0.08]	0.87	−0.08	[−0.16, −0.003]	0.04	0.002	[−0.07, 0.07]	0.96

^1^Data are presented as mean ± SD, median [IQR] or absolute number (%). Associations of biomarkers with variables were tested via univariable (model 1) and multivariable (model 2) regression analyses of which standardized β coefficients (St β) are given, referring to the number of SD change in the dependent variable (biomarker) per SD increment in the independent variable. Model 1: crude model; model 2: model 1 + adjustment for age, sex, total caloric intake, BMI, and eGFR. KTR, kidney transplant recipient; NA, not applicable; Ref, reference; st β, standardized β coefficient; uex1MH, urinary 1-methylhistidine excretion; uex3MH, urinary 3-methylhistidine excretion.

2 Maroni equation (g/d) = 0.18 * urinary urea excretion in mmol/24 h + 0.19 * body weight in kg + urinary protein excretion in g/d.

3 Data do not sum up to 100% because of missing data [*n* = 58 (8.6%)].

In the univariable model (Table 3: model 1), both uex1MH and uex3MH were significantly associated with urinary urea excretion, Maroni-calculated protein intake, FFQ-derived total protein intake, animal protein intake, and total meat intake. Uex1MH was also associated with white meat (st *β*: 0.20; 95% CI: 0.12, 0.28; *P* < 0.001) and fish intake, while uex3MH was associated with red meat intake (st *β*: 0.30; 95% CI: 0.23, 0.38; *P* < 0.001), plant protein intake, total fat intake, energy intake in men, alcohol intake, and total carbohydrate intake (Table 3: model 1). Additionally, uex3MH was inversely associated with fruit intake.

In the multivariable models (Table 3: model 2), adjustments for age, sex, energy intake, BMI, and eGFR strengthened the association of uex1MH with the Maroni-calculated protein intake, FFQ-derived total protein intake, animal protein intake, and fish intake, but weakened the association of uex1MH with total meat intake (st *β*: 0.13; 95% CI: 0.05, 0.21; *P =* 0.002 compared with st *β*: 0.11; 95% CI: 0.02, 0.19; *P* = 0.01). For uex3MH, the adjustments of model 2 weakened the associations with urea excretion, Maroni calculated protein intake, FFQ-derived total protein and animal protein intake, total meat intake, and red meat intake. Interestingly, the multivariable model unveiled a positive association of uex3MH with fish intake (st *β*: 0.07; 95% CI: 0.01, 0.14; *P* = 0.03), while the associations of uex3MH with plant protein intake and total carbohydrate intake became inverse (st *β*: −0.17; 95% CI: −0.28, −0.05; *P* = 0.004 and st *β*: −0.19; 95% CI: −0.33, −0.05; *P* = 0.01, respectively). The associations of uex3MH with fruit, total fat, and alcohol intakes were no longer significant after the adjustments in the multivariable analysis (Table 3: model 2).

### Association of meat intake biomarkers with mortality and graft failure

During median follow-up of 5.4 (IQR: 4.9–6.1) y, 145 (21%) KTR died. Of these, 60 (41%) died of cardiovascular disease, 40 (28%) of infectious causes, 23 (16%) of malignancy, 20 (14%) of miscellaneous causes, and 2 (1%) of unknown causes. Prospective analyses of the associations of log_2_-transformed uex1MH and log_2_-transformed uex3MH with mortality and death-censored graft failure are described in **Table 4**. The proportionality of hazards assumption was checked with the Schoenfeld residual test and was not violated for the associations (*P* > 0.05).

**TABLE 4:** Cox regression analyses for the associations of log_2_-transformed urinary excretions of 1-methylhistidine and 3-methylhistidine with mortality and graft failure in KTR^1^

	1-Methylhistidine		3-Methylhistidine	
	HR (95% CI)^2^	*P* value	HR (95% CI)^2^	*P* value
All-cause mortality				
Crude	0.82 (0.74, 0.91)	<0.001	0.55 (0.42, 0.72)	<0.001
Model 1	0.90 (0.80, 1.01)	0.07	0.59 (0.41, 0.83)	0.003
Model 2	0.91 (0.81, 1.03)	0.13	0.55 (0.38, 0.78)	0.001
Model 3	0.91 (0.81, 1.03)	0.14	0.59 (0.41, 0.86)	0.01
Model 4	0.89 (0.79, 1.00)	0.06	0.58 (0.41, 0.82)	0.002
Model 5	0.91 (0.80, 1.02)	0.10	0.60 (0.42, 0.87)	0.01
Model 6	0.91 (0.81, 1.04)	0.16	0.62 (0.41, 0.93)	0.02
Model 7	0.92 (0.82, 1.04)	0.19	0.65 (0.45, 0.93)	0.02
Model 8	0.90 (0.80, 1.02)	0.09	0.59 (0.41, 0.84)	0.003
Model 9	0.90 (0.80, 1.01)	0.08	0.53 (0.36, 0.79)	0.002
Graft failure				
Crude	0.84 (0.73, 0.96)	0.01	0.59 (0.41, 0.85)	0.004
Model 1	0.82 (0.69, 0.97)	0.02	0.54 (0.33, 0.88)	0.01
Model 2	0.82 (0.69, 0.99)	0.04	0.55 (0.33, 0.94)	0.03
Model 3	0.77 (0.64, 0.92)	0.01	0.50 (0.30, 0.83)	0.01
Model 4	0.81 (0.68, 0.96)	0.02	0.55 (0.34, 0.90)	0.02
Model 5	0.84 (0.70, 0.99)	0.04	0.55 (0.33, 0.91)	0.02
Model 6	0.82 (0.68, 0.99)	0.04	0.54 (0.31, 0.92)	0.02
Model 7	0.84 (0.70, 1.01)	0.06	0.58 (0.35, 0.97)	0.04
Model 8	0.81 (0.69, 0.97)	0.02	0.55 (0.33, 0.90)	0.02
Model 9	0.82 (0.69, 0.98)	0.03	0.59 (0.35, 1.00)	0.05
Crude	Log_2_-transformed variable.			
Model 1	Crude + adjustments for age, sex, BMI, eGFR, proteinuria, time from transplantation to baseline, and FFQ-estimated energy intake.			
Model 2	Model 1 + adjustments for postmortal donation, cold ischemia time, total dialysis time, total number of transplantations, primary renal disease pretransplantation.			
Model 3	Model 1 + delayed graft function, rejection up to baseline, and posttransplantation CMV infection.			
Model 4	Model 1 + adjustments for prednisolone dosage, CNI usage, and proliferation inhibitor usage.			
Model 5	Model 1 + adjustments for alcohol intake.			
Model 6	Model 1 + adjustments for CRP, HDL cholesterol, diastolic blood pressure, smoking behavior, diabetes, PTDM, and SQUASH score.			
Model 7	Model 1 + adjustment for metabolic acidosis (venous pH and venous HCO_3_).			
Model 8	Model 1 + adjustment for serum iron.			
Model 9	Model 1 + adjustment for 24-h urinary taurine excretion.			

1 *n* = 678. CMV, cytomegalovirus; CNI, calcineurin inhibitors; CRP, C-reactive protein; eGFR, estimated glomerular filtration rate; KTR, kidney transplant recipient; SQUASH, Short Questionnaire to Assess Health-enhancing physical activity.

^2^Per log_2_ increment = per doubling of urinary 1-methylhistidine or 3-methylhistidine excretion.

In univariable Cox regression analyses, uex1MH and uex3MH were both associated with significantly lower risk of mortality (HR per doubling, uex1MH: 0.82; 95% CI: 0.74, 0.91; *P* < 0.001; and uex3MH: 0.55; 95% CI: 0.42, 0.72; *P* < 0.001). The inverse association of uex1MH with mortality was lost after adjustment for potential confounders. The inverse association of uex3MH with mortality remained independent of further adjustments (models 1–9).

Of 678 KTR, 83 (12%) subjects developed graft failure. Most of these patients developed chronic rejection (*n* = 61, 74%). Other causes include vascular problems, infections, and other miscellaneous causes of graft failure. Univariable Cox regression analyses revealed an inverse association of uex1MH and uex3MH with graft failure (HR per doubling: uex1MH: 0.84; 95% CI: 0.73, 0.96; *P* = 0.01; and uex3MH: 0.59; 95% CI: 0.41, 0.85; *P =* 0.004). The association of uex1MH with lower risk of graft failure was independent of adjustments for potential confounders (models 1–6). However, when adjusted for metabolic acidosis markers, the association became borderline significant, with HR per doubling: uex1MH: 0.84; 95% CI: 0.70,1.01; *P* = 0.06 (model 7). The association of uex3MH with lower risk of graft failure was independent of adjustments for potential confounders including transplantation complications (models 1–7).

We additionally adjusted for other elements that are also abundantly found in meat. Adjusting for iron did not change the associations of uex1MH and uex3MH with outcomes (Table 4: model 8). Adjusting for urinary taurine did not materially change the association of uex1MH and uex3MH with mortality (Table 4: model 9). Also, after adjustment for taurine the association of uex1MH with graft failure did not materially change (HR per doubling: 0.82; 95% CI: 0.69, 0.98; *P* = 0.03), but did slightly weaken the association of uex3MH with graft failure [HR per doubling: 0.59; 95% CI: 0.35, 1.00; *P* = 0.05 (model 9)].

**FIGURE 1:**
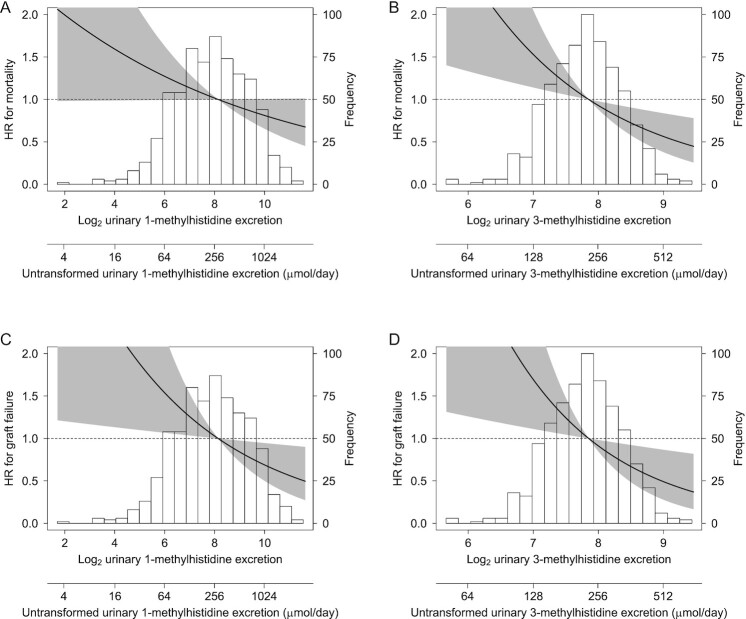
Linear splines of the associations of log_2_-transformed 24-h urinary 1-methylhistidine and 3-methylhistidine excretions with mortality and graft failure. Data were fit by a Cox proportional hazard model and were adjusted for age, sex, BMI, eGFR rate, proteinuria, time from transplantation to baseline visit, and FFQ-estimated energy intake, *n* = 678. The black line represents the HR, while the gray area represents the 95% CI. The HRs were plotted relative to a value of 1.0 for the mean value of either uex1MH or uex3MH as a reference, respectively. A histogram of each distribution is plotted in the background. Association of urinary 1-methylhistidine excretion with mortality (A), association of urinary 3-methylhistidine excretion with mortality (B), association of urinary 1-methylhistidine excretion with graft failure (C), and association of urinary 3-methylhistidine excretion with graft failure (D). eGFR, estimated glomerular filtration rate; uex1MH, urinary 1-methylhistidine excretion; uex3MH, urinary 3-methylhistidine excretion.

No significant interactions with age, sex, BMI, eGFR, or alcohol intake were found for the associations of uex1MH and uex3MH with outcomes (*P* > 0.05). Spline analyses in **Figure 1** depict the associations of log_2_ transformed uex1MH and uex3MH with mortality (A, B) and graft failure (C, D).

When excluding KTRs with a baseline eGFR <15 mL/min/1.73 m^2^ (*n* = 17), the associations of uex1MH and uex3MH with graft failure did not materially change (model 1; HR per doubling of uex1MH: 0.82; 95% CI: 0.69, 0.99; *P* = 0.03, and per doubling of uex3MH: 0.54; 95% CI: 0.32, 0.93; *P* = 0.03).

## Discussion

In the current study in KTR, we found that uex1MH is independently and significantly associated with white meat intake, while uex3MH is independently and significantly associated with red meat intake, supporting their roles as biomarkers for white and red meat, respectively. We found that uex3MH is inversely associated with mortality, and that both uex1MH and uex3MH are inversely associated with graft failure, independently of adjustments for potential confounders.

Several human studies have shown a dose-dependent increase in uex1MH and uex3MH after meat intake (11, 12, 30, 31). We observed in the current study that uex1MH is associated with specifically white meat and fish intake, and that uex3MH is associated with red meat intake, corroborating previous findings (12, 14).

When looking at the meat supply in the Western world, the red meat supply (53.9 kg/y/capita) in the Netherlands was lower, whereas the white meat supply (22.5/kg/y/capita) supply was higher than that in Germany. The supply of both kinds of meat was higher in the United States than in the Netherlands at the time of study inclusion (32).

A major finding of this study is the inverse association of uex1MH with graft failure. This finding suggests that high intake of white meat is protective for allograft outcome in KTR. This outcome may in part be explained by an improvement of nutritional status (33). Earlier, we found that high protein intake is associated with improved patient and graft survival in KTR (3, 4). KTR may be at risk of protein energy wasting, partially because of the constant low-grade inflammation reaction against the allograft and partially because of corticosteroid-related protein catabolism (34, 35). High intake of protein, especially white meat, may in part compensate for protein energy wasting in KTR, resulting in favorable graft outcomes (4). Second, the inverse association of uex1MH with graft failure may be explained in part by its origin. Uex1MH largely originates from the metabolism of dietary anserine through poultry intake. Anserine is endowed with a broad spectrum of biological properties, including antioxidant and quenching effects (36, 37). Studies suggest that short-term treatment with anserine improved vascular permeability and proteinuria in diabetic mice (37). Anserine and other histidine-containing peptides are mobile cytoplasmic buffers that facilitate the exchange of ions such as H^+^, acting as biological pumps, in circumstances of acidobasic imbalances (38, 39). Thus, it is plausible that these mechanisms might indirectly mediate the protective association of uex1MH with graft failure.

Another major finding of this study is the inverse and independent association of uex3MH with mortality and graft failure. Also, we found that specific transplantation-related determinants of graft loss (40), such as HLA mismatches and immunosuppression, had minimal influence on the prospective association of uex1MH and uex3MH with graft failure. Our results suggest that red meat intake is protective against graft failure in this population. Meat is an important nutritional source of functional amino acids and dipeptides (41), and the renoprotective properties derived from these (42, 43) might be of high relevance considering the inflammatory milieu that might take place in the kidney of KTR. Furthermore, because histidine-containing peptides and taurine also promote skeletal muscle health (44, 45), it is likely that they also contribute in preventing protein energy wasting in KTR.

Of note, adjustment for urinary taurine excretion did slightly weaken the association of uex3MH with graft failure. This does not necessarily mitigate the suggestion that the association of uex3MH is fueled by dietary meat intake, as taurine excretion also reflects meat intake and was shown to be inversely associated with graft failure in the past (28).

Some studies in the general population suggest that high red meat intake is associated with adverse outcomes, including kidney disease and kidney failure (5–7, 46–48), while studies in patients with a higher likelihood of underlying CKD, particularly patients with type 2 diabetes, are more suggestive of a protective effect. As such, in the ONTARGET (Ongoing Telmisartan Alone and in combination with Ramipril Global Endpoint Trial) study, animal protein intake was prospectively associated with lower risk of development or progression of CKD among these patients (49). In line with these findings, the American Diabetes Association does not recommend restricting protein intake in patients with diabetes or diabetic kidney disease (50), given the higher risk of malnutrition that protein restriction might pose in these patients (51). Our study results are also in line with these findings by suggesting that intake of meat, including red meat, is beneficial for long-term kidney survival in KTR. A possible explanation is that high red meat intake may partially compensate for the previously mentioned risk of protein energy wasting in KTR (3, 4). Another possible explanation is that meat intake, as a part of animal protein intake, can have specific advantages. As such, meat intake is generally of high protein quality and digestibility and has superior bioavailability of important physiological elements (41, 52, 53). Altogether, these properties in meat might indirectly explain the beneficial effects of meat on graft survival in KTR.

In the Lifelines Cohort Study of the general population in the Netherlands, animal protein intake, in particular meat, fish, and egg intake, was positively associated with muscle mass, but plant protein intake was not (54). Interestingly, this association was strongest in elderly women (age >65 y), which supports the growing belief that older individuals should increase their protein intake, possibly through increased meat intake, above the recommended daily allowance to prevent wasting (55). This may also apply for the current study, given the median age of 55 (range: 45–63) y, implying that 25% of the study population is older than 63 y. It should be noted that high red meat intake is associated with other adverse outcomes (e.g., colon carcinoma and hypertension) (56, 57). Future intervention studies should also take these outcomes into account.

Strengths of this study are its large sample size, no loss to follow-up, minimal missing data, the ability to measure uex1MH and uex3MH in 24-h urine samples to account for daily dietary changes, and the comparison of these meat intake biomarkers with well-established total protein intake biomarkers, i.e., urea excretion and with data derived from the FFQ. It must be noted, however, that FFQ data is often biased by underreporting, especially for total protein intake (58). A limitation of the study design is the use of a single collection moment for 24-h urine, which can result in bias through day-to-day variation of specific protein intake. Another limitation is that adjustment for other trace elements, including, e.g., zinc, was not possible because these data were not available.

Of note, the mean eGFR of ∼45 mL/min/1.73 m^2^ in the KTR from our cohort is lower than the mean eGFR of 50–55 mL/min/1.73 m^2^ at 1 y after transplantation reported for the United States (59). For this difference, it may be relevant to consider the context of maintenance immunosuppression. In the US report, the majority (67–93%) of KTR were receiving tacrolimus-based maintenance immunosuppression, while a minority (3–27%) were receiving cyclosporine-based maintenance immunosuppression. In our cohort, 39% of patients were on cyclosporine-based maintenance immunosuppression, while 18% were on tacrolimus-based maintenance immunosuppression (60). In a cohort based on the FAVORIT (Folic Acid for Vascular Outcome Reduction in Transplantation) trial (61), also with higher use of cyclosporine-based than tacrolimus-based maintenance immunosuppression (51% compared with 38%), mean eGFR was ∼49 mL/min/1.73 m^2^. So, it may be considered a limitation of our study that mean eGFR value and use of tacrolimus-based immunosuppression were relatively low. It would be relevant to replicate our findings in more contemporary cohorts with a higher mean eGFR value and higher use of tacrolimus-based immunosuppression.

In conclusion, we found that high excretions of uex1MH as a biomarker of white meat intake and uex3MH as a biomarker of red meat intake are associated with lower risk of graft failure in KTR. These associations may be explained through potential benefits of white and red meat intake and through potential compensation of protein energy wasting in KTR, although further studies are required to confirm this. Future intervention studies using these biomarkers are warranted to study the effect of high meat intake on graft failure in KTR.

## Supplementary Material

nqab185_Supplemental_File

## Data Availability

Data described in the manuscript, code book, and analytic code will be made available upon request pending.

## Notes

MYS and ARN contributed equally to this work.

This study was funded by the Top Institute Food and Nutrition of the Netherlands (grant A-1003). The funding organization is a nongovernmental entity. It was not involved in the design, implementation, analysis, or interpretation of the data.

Supplemental Figure 1 and Supplemental Table 1 are available from the “Supplementary data” link in the online posting of the article and from the same link in the online table of contents at https://academic.oup.com/ajcn/.

CKD, chronic kidney disease; CNI, calcineurin inhibitor; CRP, C-reactive protein; eGFR, estimated glomerular filtration rate; GFR, glomerular filtration rate; HbA1c, glycated hemoglobin; HLA, human leukocyte antigen; KTR, kidney transplant recipient; NT-proBNP, N-terminal pro brain natriuretic peptide; PTDM, posttransplantation diabetes mellitus; SQUASH, Short Questionnaire to Assess Health-enhancing physical activity; st *β*, standardized *β*; TIA, transient ischemic attack; uex1MH, urinary 1-methylhistidine excretion; uex3MH, urinary 3-methylhistidine excretion.

## References

[1] Ojo AO, Hanson JA, Wolfe RA, Leichtman AB, Agodoa LY, Port FK. Long-term survival in renal transplant recipients with graft function. Kidney Int. 2000;57(1):307–13. 10620213 10.1046/j.1523-1755.2000.00816.x

[2] Meier-Kriesche H-U, Schold JD, Srinivas TR, Kaplan B. Lack of improvement in renal allograft survival despite a marked decrease in acute rejection rates over the most recent era. Am J Transplant. 2004;4(3):378–83. 14961990 10.1111/j.1600-6143.2004.00332.x

[3] Deetman PE, Said MY, Kromhout D, Dullaart RPF, Kootstra-Ros JE, Sanders JSF, Seelen MAJ, Gans ROB, Navis G, Joosten MM et al. Urinary urea excretion and long-term outcome after renal transplantation. Transplantation. 2015;99(5):1009–15. 25393159 10.1097/TP.0000000000000464

[4] Said MY, Deetman PE, de Vries APJ, Zelle DM, Gans ROB, Navis G, Joosten MM, Bakker SJL. Causal path analyses of the association of protein intake with risk of mortality and graft failure in renal transplant recipients. Clin Transplant. 2015;29(5):447–57. 25739949 10.1111/ctr.12536

[5] Sinha R, Cross AJ, Graubard BI, Leitzmann MF, Schatzkin A. Meat intake and mortality: a prospective study of over half a million people. Arch Intern Med. 2009;169(6):562–71. 19307518 10.1001/archinternmed.2009.6 PMC2803089

[6] Haring B, Selvin E, Liang M, Coresh J, Grams ME, Petruski-Ivleva N, Steffen LM, Rebholz CM. Dietary protein sources and risk for incident chronic kidney disease: results from the Atherosclerosis Risk in Communities (ARIC) study. J Ren Nutr. 2017;27(4):233–42. 28065493 10.1053/j.jrn.2016.11.004 PMC5476496

[7] Lew QLJ, Jafar TH, Koh HWL, Jin A, Chow KY, Yuan JM, Koh WP. Red meat intake and risk of ESRD. J Am Soc Nephrol. 2017;28(1):304–12. 27416946 10.1681/ASN.2016030248 PMC5198288

[8] Pijls LTJ, de Vries H, Donker AJM, van Eijk JTM. Reproducibility and biomarker-based validity and responsiveness of a food frequency questionnaire to estimate protein intake. Am J Epidemiol. 1999;150(9):987–95. 10547145 10.1093/oxfordjournals.aje.a010108

[9] Molag ML, de Vries JHM, Ocké MC, Dagnelie PC, van den Brandt PA, Jansen M, van Staveren WA, van ’t Veer P. Design characteristics of food frequency questionnaires in relation to their validity. Am J Epidemiol. 2007;166(12):1468–78. 17881382 10.1093/aje/kwm236

[10] Crush KG. Carnosine and related substances in animal tissues. Comp Biochem Physiol. 1970;34(1):3–30. 4988625 10.1016/0010-406x(70)90049-6

[11] Sjölin J, Hjort G, Friman G, Hambraeus L. Urinary excretion of 1-methylhistidine: a qualitative indicator of exogenous 3-methylhistidine and intake of meats from various sources. Metabolism. 1987;36(12):1175–84. 3683186 10.1016/0026-0495(87)90245-9

[12] Abe H, Okuma E, Sekine H, Maeda A, Yoshiue S. Human urinary excretion of L-histidine-related compounds after ingestion of several meats and fish muscle. Int J Biochem. 1993;25:1245–9. 8224369 10.1016/0020-711x(93)90074-o

[13] Mitry P, Wawro N, Rohrmann S, Giesbertz P, Daniel H, Linseisen J. Plasma concentrations of anserine, carnosine and pi-methylhistidine as biomarkers of habitual meat consumption. Eur J Clin Nutr. 2019;73(5):692–702. 30018457 10.1038/s41430-018-0248-1

[14] Cross AJ, Major JM, Sinha R. Urinary biomarkers of meat consumption. Cancer Epidemiol Biomarkers Prev. 2011;20(6):1107–11. 21527577 10.1158/1055-9965.EPI-11-0048 PMC3111815

[15] Asatoor AM, Armstrong MD. 3-Methylhistidine, a component of actin. Biochem Biophys Res Commun. 1967;26(2):168–74. 6067661 10.1016/0006-291x(67)90229-x

[16] Johnson P, Harris CI, Perry SV. 3-methylhistidine in actin and other muscle proteins. Biochem J. 1967;105(1):361–70. 6056634 10.1042/bj1050361 PMC1198308

[17] Long CL, Haverberg LN, Young VR, Kinney JM, Munro HN, Geiger JW. Metabolism of 3-methylhistidine in man. Metabolism. 1975;24(8):929–35. 1143090 10.1016/0026-0495(75)90084-0

[18] Altorf-van der Kuil W, Brink EJ, Boetje M, Siebelink E, Bijlsma S, Engberink MF, Veer PVT, Tomé D, Bakker SJL, van Baak MA et al. Identification of biomarkers for intake of protein from meat, dairy products and grains: a controlled dietary intervention study. Br J Nutr. 2013;110(5):810–22. 23452466 10.1017/S0007114512005788

[19] van den Berg E, Engberink MF, Brink EJ, van Baak MA, Gans ROB, Navis G, Bakker SJL. Dietary protein, blood pressure and renal function in renal transplant recipients. Br J Nutr. 2013;109(8):1463–70. 22906209 10.1017/S0007114512003455

[20] Snijder PM, van den Berg E, Whiteman M, Bakker SJL, Leuvenink HGD, van Goor H. Emerging role of gasotransmitters in renal transplantation. Am J Transplant. 2013;13(12):3067–75. 24266966 10.1111/ajt.12483

[21] van den Berg E, Pasch A, Westendorp WH, Navis G, Brink EJ, Gans ROB, van Goor H, Bakker SJL. Urinary sulfur metabolites associate with a favorable cardiovascular risk profile and survival benefit in renal transplant recipients. J Am Soc Nephrol. 2014;25(6):1303–12. 24511127 10.1681/ASN.2013050497 PMC4033367

[22] Wendel-Vos GCW, Schuit AJ, Saris WHM, Kromhout D. Reproducibility and relative validity of the short questionnaire to assess health-enhancing physical activity. J Clin Epidemiol. 2003;56(12):1163–9. 14680666 10.1016/s0895-4356(03)00220-8

[23] Mallon DH, Summers DM, Bradley JA, Pettigrew GJ. Defining delayed graft function after renal transplantation: simplest is best. Transplantation. 2013;96(10):885–9. 24056620 10.1097/TP.0b013e3182a19348

[24] van den Berg E, Engberink MF, Brink EJ, van Baak MA, Joosten MM, Gans ROB, Navis G, Bakker SJL. Dietary acid load and metabolic acidosis in renal transplant recipients. Clin J Am Soc Nephrol. 2012;7(11):1811–8. 22935845 10.2215/CJN.04590512 PMC3488949

[25] Siebelink E, Geelen A, De Vries JHM. Self-reported energy intake by FFQ compared with actual energy intake to maintain body weight in 516 adults. Br J Nutr. 2011;106(2):274–81. 21338536 10.1017/S0007114511000067

[26] Voedingscentrum Den Haag, Stichting Nederlands Voedingsstoffenbestand Zeist. NEVO-tabel: Nederlands Voedingsstoffenbestand 2006.

[27] Maroni BJ, Steinman TI, Mitch WE. A method for estimating nitrogen intake of patients with chronic renal failure. Kidney Int. 1985;27(1):58–65. 3981873 10.1038/ki.1985.10

[28] Post A, Said MY, Gomes-Neto AW, van der Krogt J, de Blaauw P, Berger SP, Geleijnse JM, Borgonjen K, van den Berg E, van Goor H et al. Urinary taurine excretion and risk of late graft failure in renal transplant recipients. Nutrients. 2019;11(9):2212. 10.3390/nu11092212 PMC6770760 31540245

[29] Inker LA, Eckfeldt J, Levey AS, Leiendecker-Foster C, Rynders G, Manzi J, Waheed S, Coresh J. Expressing the CKD-EPI (Chronic Kidney Disease Epidemiology Collaboration) cystatin C equations for estimating GFR with standardized serum cystatin C Values. Am J Kidney Dis. 2011;58(4):682–4. 21855190 10.1053/j.ajkd.2011.05.019 PMC4421875

[30] Datta SP, Harris H. Dietary origin of urinary methyl-histidine. Nature. 1951;168(4268):296–7. 14875074 10.1038/168296a0

[31] Dragsted LO. Biomarkers of meat intake and the application of nutrigenomics. Meat Sci. 2010;84(2):301–7. 20374789 10.1016/j.meatsci.2009.08.028

[32] Food and Agriculture Organization of the United Nations Statistics Division. Food balance sheets. Rome (Italy): FAO Statistics;2009.

[33] Lee SW, Kim YS, Kim YH, Chung W, Park SK, Choi KH, Ahn C, Oh KH. Dietary protein intake, protein energy wasting, and the progression of chronic kidney disease: analysis from the Know-CKD study. Nutrients. 2019;11(1):121. 10.3390/nu11010121 PMC6356719 30626166

[34] Martins C, Pecoits-Filho R, Riella MC. Nutrition for the post-renal transplant recipients. Transplant Proc. 2004;36(6):1650–4. 15350441 10.1016/j.transproceed.2004.06.065

[35] Kalantar-Zadeh K, Cano NJ, Budde K, Chazot C, Kovesdy CP, Mak RH, Mehrotra R, Raj DS, Sehgal AR, Stenvinkel P et al. Diets and enteral supplements for improving outcomes in chronic kidney disease. Nat Rev Nephrol. 2011;7(7):369–84. 21629229 10.1038/nrneph.2011.60 PMC3876473

[36] Boldyrev AA, Aldini G, Derave W. Physiology and pathophysiology of carnosine. Physiol Rev. 2013;93:4, 1803–45. 24137022 10.1152/physrev.00039.2012

[37] Peters V, Calabrese V, Forsberg E, Volk N, Fleming T, Baelde H, Weigand T, Thiel C, Trovato A, Scuto M et al. Protective actions of anserine under diabetic conditions. Int J Mol Sci. 2018;19(9):2751. 10.3390/ijms19092751 PMC6164239 30217069

[38] Swietach P, Youm JB, Saegusa N, Leem CH, Spitzer KW, Vaughan-Jones RD. Coupled Ca2+/H+ transport by cytoplasmic buffers regulates local Ca2+ and H+ ion signaling. Proc Natl Acad Sci. 2013;110(22):E2064–73. 23676270 10.1073/pnas.1222433110 PMC3670334

[39] Swietach P, Leem CH, Spitzer KW, Vaughan-Jones RD. Pumping Ca2+ up H+ gradients: a Ca2+-H+ exchanger without a membrane. J Physiol. 2014;592(15):3179–88. 24514908 10.1113/jphysiol.2013.265959 PMC4146368

[40] Kaboré R, Haller MC, Harambat J, Heinze G, Leffondré K. Risk prediction models for graft failure in kidney transplantation: a systematic review. Nephrology Dialysis Transplantation. 2017;32(suppl_2):ii68–76. 10.1093/ndt/gfw405 28206633

[41] Wu G. Important roles of dietary taurine, creatine, carnosine, anserine and 4-hydroxyproline in human nutrition and health. Amino Acids. 2020;52(3):329–60. 32072297 10.1007/s00726-020-02823-6 PMC7088015

[42] Kim C, Choi HS, Kim JW. Taurine chloramine inhibits the production of nitric oxide and superoxide anion by modulating specific mitogen-activated protein kinases. Adv Exp Med Biol. 2006;583:493–8. 17153636 10.1007/978-0-387-33504-9_55

[43] Marcinkiewicz J, Kurnyta M, Biedroń R, Bobek M, Kontny E, Maśliński W. Anti-inflammatory effects of taurine derivatives (taurine chloramine, taurine bromamine, and taurolidine) are mediated by different mechanisms. Adv Exp Med Biol. 2006;586:481–92. 10.1007/978-0-387-33504-9_54 17153635

[44] Maemura H, Goto K, Yoshioka T, Sato M, Takahata Y, Morimatsu F, Takamatsu K. Effects of carnosine and anserine supplementation on relatively high intensity endurance performance. Int J Sport Health Sci. 2006;4:86–94.

[45] De Luca A, Pierno S, Camerino DC. Taurine: the appeal of a safe amino acid for skeletal muscle disorders. J Transl Med. 2015;13(1):423. 10.1186/s12967-015-0610-1 PMC4513970 26208967

[46] Latkin CA, Edwards C, Davey-Rothwell MA, Tobin KE. The relationship between social desirability bias and self-reports of health, substance use, and social network factors among urban substance users in Baltimore, Maryland. Addict Behav. 2017;73:133–6. 28511097 10.1016/j.addbeh.2017.05.005 PMC5519338

[47] Lin J, Hu FB, Curhan GC. Associations of diet with albuminuria and kidney function decline. Clin J Am Soc Nephrol. 2010;5(5):836–43. 20299364 10.2215/CJN.08001109 PMC2863979

[48] Lin J, Fung TT, Hu FB, Curhan GC. Association of dietary patterns with albuminuria and kidney function decline in older white women: a subgroup analysis from the Nurses Health Study. Am J Kidney Dis. 2011;57(2):245–54. 21251540 10.1053/j.ajkd.2010.09.027 PMC3026604

[49] Dunkler D, Kohl M, Teo KK, Heinze G, Dehghan M, Clase CM, Gao P, Yusuf S, Mann JFE, Oberbauer R. Dietary risk factors for incidence or progression of chronic kidney disease in individuals with type 2 diabetes in the European Union. Nephrol Dial Transplant. 2015; 30(Suppl 4): iv76–85. 26209742 10.1093/ndt/gfv086

[50] Evert AB, Boucher JL, Cypress M, Dunbar SA, Franz MJ, Mayer-Davis EJ, Neumiller JJ, Nwankwo R, Verdi CL, Urbanski P et al. Nutrition therapy recommendations for the management of adults with diabetes. Diabetes Care. 2013;36(11):3821–42. 24107659 10.2337/dc13-2042 PMC3816916

[51] Meloni C, Morosetti M, Suraci C, Pennafina MG, Tozzo C, Taccone-Gallucci M, Casciani CU. Severe dietary protein restriction in overt diabetic nephropathy: benefits or risks?. J Ren Nutr. 2002;12(2):96–101. 11953922 10.1053/jren.2002.31762

[52] Berrazaga I, Micard V, Gueugneau M, Walrand S. The role of the anabolic properties of plant- versus animal-based protein sources in supporting muscle mass maintenance: a critical review. Nutrients. 2019;11(8):1825. 10.3390/nu11081825 PMC6723444 31394788

[53] Elango R, Levesque C, Ball RO, Pencharz PB. Available versus digestible amino acids—new stable isotope methods. Br J Nutr. 2012;108(S2):S306–14. 23107543 10.1017/S0007114512002498

[54] Alexandrov NV, Eelderink C, Singh-Povel CM, Navis GJ, Bakker SJL, Corpeleijn E. Dietary protein sources and muscle mass over the life course: the Lifelines cohort study. Nutrients. 2018;10(10):1471. 10.3390/nu10101471 PMC6212815 30308987

[55] Bauer J, Biolo G, Cederholm T, Cesari M, Cruz-Jentoft AJ, Morley JE, Phillips S, Sieber C, Stehle P, Teta D et al. Evidence-based recommendations for optimal dietary protein intake in older people: a position paper from the PROT-AGE Study Group. J Am Med Dir Assoc. 2013;14(8):542–59. 23867520 10.1016/j.jamda.2013.05.021

[56] Aykan NF. Red meat and colorectal cancer. Oncol Rev. 2015:9(1):288. 26779313 10.4081/oncol.2015.288 PMC4698595

[57] Griep LMO, Seferidi P, Stamler J, Van Horn L, Chan Q, Tzoulaki I, Steffen LM, Miura K, Ueshima H, Okuda N et al. Relation of unprocessed, processed red meat and poultry consumption to blood pressure in East Asian and Western adults. J Hypertens. 2016;34(9):1721–9. 27379533 10.1097/HJH.0000000000001008 PMC6524524

[58] Slimani N, Bingham S, Runswick S, Ferrari P, Day NE, Welch AA, Key TJ, Miller AB, Boeing H, Sieri S et al. Group level validation of protein intakes estimated by 24-hour diet recall and dietary questionnaires against 24-hour urinary nitrogen in the European Prospective Investigation into Cancer and Nutrition (EPIC) calibration study. Cancer Epidemiol Biomarkers Prev. 2003;12(8):784–95. 12917211

[59] Huang Y, Tilea A, Gillespie B, Shahinian V, Banerjee T, Grubbs V, Powe N, Rios-Burrows N, Pavkov M, Saran R. Understanding trends in kidney function 1 year after kidney transplant in the United States. J Am Soc Nephrol. 2017;28(8):2498–510. 28270413 10.1681/ASN.2016050543 PMC5533222

[60] Minović I, Riphagen IJ, Van Den Berg E, Kootstra-Ros JE, Van Faassen M, Gomes Neto AW, Geleijnse JM, Gans RO, Eggersdorfer M, Navis GJ et al. Vitamin B-6 deficiency is common and associated with poor long-term outcome in renal transplant recipients. Am J Clin Nutr. 2017;105(6):1344–50. 28468895 10.3945/ajcn.116.151431

[61] Weiner DE, Park M, Tighiouart H, Joseph AA, Carpenter MA, Goyal N, House AA, yuan H C, Ix JH, Jacques PF et al. Albuminuria and allograft failure, cardiovascular disease events, and all-cause death in stable kidney transplant recipients: a cohort analysis of the FAVORIT Trial. Am J Kidney Dis. 2019;73(1):51–61. 30037726 10.1053/j.ajkd.2018.05.015 PMC6309643

