# Supplementary material for: Meat intake and risk of mortality and graft failure in kidney transplant recipients
Source: Am J Clin Nutr. 2021 Jun 5;114(4):1505–17. doi: 10.1093/ajcn/nqab185 (PMC8488867; doi:10.1093/ajcn/nqab185)
Supplement: nqab185_Supplemental_File [file nqab185_supplemental_file.docx]

**Supplementary data**

**To the manuscript “Meat intake and risk of mortality and graft failure in kidney transplant recipients”; M. Said et. al.**

| Supplementary Table 1. Food frequency questionnaire meat intake combinations. | | | |
| --- | --- | --- | --- |
| *Red meat* | *White meat* | *Meat and meat products* | *Fish* |
| Beef: beefsteak and other types of whole meat beef meat products | Chicken and chicken products | Chicken and chicken products | Raw herring and herring snack |
| Beef: ‘blinde vink’* and other types of processed beef products | Turkey and turkey products | Turkey and turkey products | Salmon and similar fish |
| Pork: pork leg meat and other types of boneless whole pork meat products |  | Beef: beefsteak and other types of whole meat beef meat products | Flounder and similar flatfish |
| Pork: pork chops and other types of whole pork meat products with bone |  | Beef: ‘blinde vink’ and other types of processed beef products | Trout, plaice, gurnard, and other types of fish |
| Pork: smoked sausage and other types of processed pork products |  | Pork: pork leg meat and other types of boneless whole pork meat products | Readymade fish |
| Processed meat products: bacon and similar meat products |  | Pork: pork chops and other types of whole pork meat products with bone | Unknown type of fish |
| Processed meat products: liver pate and similar meat products |  | Pork: smoked sausage and other types of processed pork products | Shellfish |
| Processed meat products: ham and similar products |  | Processed meat products: bacon and similar meat products |  |
| Processed meat products: bologna sausage and similar products |  | Processed meat products: liver pate and similar meat products |  |
| Processed meat products: snack sausage and similar products |  | Processed meat products: ham and similar products |  |
| Cooked liver |  | Processed meat products: bologna sausage and similar products |  |
| Liver and kidney products |  | Processed meat products: snack sausage and similar products |  |
| Lamb or sheep meat |  | Cooked liver |  |
| Ground meats (all sorts) |  | Liver and kidney products |  |
| Other meat products: goat, horse, etc. |  | Lamb or sheep meat |  |
| Unknown meat and meat products |  | Minced meats (all sorts) |  |
|  |  | Other meat products: goat, horse, etc. |  |
|  |  | Unknown meat and meat products |  |
| * Blinde vink is a roulade-type of ground meat product, popular in the Netherlands. | | | |

**Supplementary Figure 1. Flow diagram of participant inclusion**
